# Recovery of Heat Treated *Bacillus cereus* Spores Is Affected by Matrix Composition and Factors with Putative Functions in Damage Repair

**DOI:** 10.3389/fmicb.2016.01096

**Published:** 2016-07-18

**Authors:** Alicja K. Warda, Marcel H. Tempelaars, Tjakko Abee, Masja N. Nierop Groot

**Affiliations:** ^1^TI Food and NutritionWageningen, Netherlands; ^2^Laboratory of Food Microbiology, Wageningen UniversityWageningen, Netherlands; ^3^Wageningen UR Food & Biobased ResearchWageningen, Netherlands

**Keywords:** germination and outgrowth, thermal treatment, food matrix, spore former, rice, recovery of spores

## Abstract

The ability of spores to recover and grow out after food processing is affected by cellular factors and by the outgrowth conditions. In the current communication we studied the recovery and outgrowth of individually sorted spores in BHI and rice broth media and on agar plates using flow cytometry. We show that recovery of wet heat treated *Bacillus cereus* ATCC 14579 spores is affected by matrix composition with highest recovery in BHI broth or on rice agar plates, compared to BHI agar plates and rice broth. Data show that not only media composition but also its liquid or solid state affect the recovery of heat treated spores. To determine the impact of factors with putative roles in recovery of heat treated spores, specific genes previously shown to be highly expressed in outgrowing heat-treated spores were selected for mutant construction. Spores of nine *B. cereus* ATCC 14579 deletion mutants were obtained and their recovery from wet heat treatment was evaluated using BHI and rice broth and agar plates. Deletion mutant spores showed different capacity to recover from heat treatment compared to wild type with the most pronounced effect for a mutant lacking BC5242, a gene encoding a membrane protein with C2C2 zinc finger which resulted in over 95% reduction in recovery compared to the wild type in BHI broth. Notably, similar relative performance of wild type and mutants was observed using the other recovery conditions. We obtained insights on the impact of matrix composition and state on recovery of individually sorted heat treated spores and identified cellular factors with putative roles in this process. These results may provide leads for future developments in design of more efficient combined preservation treatments.

## Introduction

An increased demand for food with improved freshness, sensorial, and nutritional values has directed food processing toward the use of milder heat treatments that require secondary mild preservation hurdles to assure stability and safety of the products (Pasha et al., 2014). As a result, these products are challenged by resistant microbial spores, that survive heat and other preservation hurdles used in food processing (Postollec et al., 2012; Stecchini et al., 2013; Checinska et al., 2015). Reduction of the heat treatment intensity may lead to subpopulations of spores that are sublethally damaged rather than inactivated resulting in increased heterogeneity in the population (Cazemier et al., 2001; Warda et al., 2015). Damaged spores retain the capacity to germinate, repair, and eventually grow out leading to spoilage and safety issues (Smelt et al., 2008; Mafart et al., 2010; Samapundo et al., 2014; Warda et al., 2015). Heterogeneity in spore populations can originate from differences in sensitivity of individual spores to inactivating treatments (Stringer et al., 2011) and/or from differences in repair capacity of individual damaged spores. In addition, the presence of superdormant spores may further increase heterogeneity (Ghosh and Setlow, 2010), and this conceivably results in less accurate prediction of spore outgrowth behavior.

Wet heat treatment is a common practice in food processing intended to reduce the microbial load of food products. Thermal pasteurization processes aim for inactivation of vegetative cells but are insufficient to kill spores. Sterilization processes aim for spore inactivation but may result in spore damage when target process conditions are not reached or when products contain highly heat resistant spores. The exact mechanism of wet heat killing of the spores and concomitant wet heat damage are not yet fully understood. Wet heat resistance of spores, mainly investigated in *Bacillus subtilis*, is determined by a number of factors including the spore structural components [small acid-soluble proteins (SASP), dipicolinic acid (DPA), metal ions, low core water content] but also the sporulation conditions (temperature, liquid or solid state of medium) affect its resistance (Setlow, 2014; Berendsen et al., 2016; Wells-Bennik et al., 2016). Wet heat treatment is thought to kill spores by damaging one or more key spore proteins, however, the identity of those proteins remains to be determined (Setlow, 2014). Analysis of single wet heat treated spores of *Clostridium botulinum* (Stringer et al., 2011) and *Bacillus* species (Wang et al., 2011) revealed a delayed initiation of germination and/or reduced rate of germination, but also the subsequent outgrowth was delayed indicating not only damage to the germination system but also to other spore components affecting outgrowth. The time required for germination and outgrowth of spores was shown to correlate with the wet heat treatment intensity (Aguirre et al., 2012). Heterogeneity in germination and outgrowth of surviving *C. botulinum* and *Bacillus cereus* spore populations is more pronounced in the presence of a secondary mild stress factor such as low pH without and with sorbic acid, and increasing levels of salt (Stringer et al., 2011; van Melis et al., 2014; Warda et al., 2015) or the natural components of food media (Warda et al., 2015). In general, damaged spores were shown to be more sensitive to secondary stresses including sodium chloride, pH, sorbic acid compared to undamaged spores (Feeherry et al., 1987; Faille et al., 1997; Cazemier et al., 2001; Cortezzo et al., 2004; Samapundo et al., 2014; van Melis et al., 2014; Warda et al., 2015, 2016). Some studies suggest a pre-plating recovery step in optimal (perhaps strain and treatment specific) conditions to allow recovery of injured cells (Wu, 2008) or spores (Rodriguez-Palacios and Lejeune, 2011). For example, a 7 h incubation step of heat treated *Clostridium difficile* spores in BHI broth prior to plating resulted in increased recovery of ethanol resistant fraction (dormant spores) on blood agar (Rodriguez-Palacios and Lejeune, 2011).

In this study we focus on *B. cereus*, a spore former of concern in processed foods. Its spores are widely present in the environment and are common contaminants in the food chain. *B. cereus* has been associated with food spoilage (Andersson et al., 1995) and food-borne disease (Stenfors Arnesen et al., 2008). The vegetative cells of *B. cereus* can cause disease either by secretion of enterotoxins in the small intestine, causing the diarrheic syndrome or by the production of a heat-stable toxin (cereulide) in food before ingestion resulting in an emetic syndrome. *B. cereus* associated diseases are usually mild and self-limiting but in rare instances they can lead to fatal outcomes (Ehling-Schulz et al., 2004; Dierick et al., 2005; Granum, 2005; Schoeni and Wong, 2005; Stenfors Arnesen et al., 2008).

Using a transcriptome approach, we previously identified 21 genes putatively involved in heat damage repair in *B. cereus.* For one of these candidate genes, *cdnL* (now referred as *cdnL1*), a role in spore damage repair was further confirmed using a targeted deletion mutant (Warda et al., 2016). Here we report on behavior of eight newly and one previously (Warda et al., 2016) constructed mutant to assess respective putative roles in recovery efficiency of heat treated *B. cereus* spores.

To this end, *B. cereus* ATCC 14579 wild type and its mutant derivative spores were exposed to a wet heat treatment resulting in over 95% of damaged spores in the surviving fractions. The recovery and outgrowth of spores was followed using flow cytometry (FCM) in combination with single spore sorting and a Most Probable Number (MPN) approach. To quantify the effect of matrix conditions on recovery capacity of wild type and mutant spores BHI and rice media, both in solid and liquid form were included. This approach allows for identification of candidate genes that may contribute to recovery capacity of heat treated *B. cereus* spores.

## Materials and Methods

### Strains and Sporulation Conditions

*Bacillus cereus* ATCC 14579 obtained from the American Type Culture Collection (ATCC), and its mutant derivatives used in this study (**Table 1**) were cultured in Bacto Brain Hart Infusion broth (BHI, Beckton Dickinson) at 30°C with aeration at 200 rpm. A nutrient-rich, chemically defined sporulation medium (MSM medium) described previously (Garcia et al., 2010) was used to obtain spores. Sporulation and spore handling were performed as described previously (Warda et al., 2015), briefly one ml of an overnight-grown pre-culture was used to inoculate 100 ml of MSM media in 500 ml flasks and incubated at 30°C with aeration at 200 rpm. Sporulation was monitored by phase contrast microscopy until over 99% of the spores were released from the mother cell (typically after 2–3 days). Released spores were harvested by centrifugation at 5,000 rpm at 4°C (5804R, Eppendorf, Germany) for 15 min and washed with chilled phosphate buffer (100 mM, pH 7.4) containing 0.1% Tween80 to prevent spore clumping. Spores were washed twice a day for 2 weeks with a phosphate buffer that was gradually decreased in Tween80 concentration until a final concentration of 0.01% (further referred as suspension buffer). Spores free of vegetative cells, debris and mother cells residues were stored at 4°C and used within 6 months. A single spore crop per strain was used for all the experiments.

**Table 1:** Overview of *Bacillus cereus* ATCC 14579 deletion mutants used in this study.

Strain/Genotype	Sorting	Function	Reference
*B. cereus* ATCC 14579 (wild type)	+		
ΔBC0460	+	Hypothetical protein	This study
ΔBC0690	+	PbsX family transcriptional regulator	This study
ΔBC0852	+	Quaternary ammonium compound-resistance protein/SugE	This study
ΔBC0853	+	Quaternary ammonium compound-resistance protein/SugE	This study
ΔBC1312	-^a^	3-hydroxybutyryl-CoA dehydratase	This study
ΔBC1314	+	PhaQ/PadR family transcriptional regulator	This study
ΔBC3437	-^b^	Cytoplasmic protein	This study
ΔBC3921	-^b^	Hypothetical protein	This study
ΔBC4834	-^a^	ArsR family transcriptional regulator	This study
ΔBC5242	+	Membrane protein with C2C2 zinc finger	This study
Δ*cdnL1* (BC4714), Cm^r^	+	CarD_CdnL_TRCF family transcriptional regulator	Warda et al., 2016
Δ*cdnL2* (BC3648)	+	CarD_CdnL_TRCF family transcriptional regulator	This study
Δ*cdnL1* (BC4714), Δ*cdnL2* (BC3648), Cm^r^	+		This study

*^a^no sporulation, ^b^poor spore quality, *Cm^*r*^* chloramphenicol resistance.*

### Construction of Deletion Mutants

Deletion mutants (**Table 1**) were constructed using the temperature-sensitive suicide plasmid pAUL-A (Chakraborty et al., 1992). Flanking regions of the individual genes were amplified using KAPA HiFi Hotstart ReadyMix (KAPA Biosystems, USA) and the primers UP_enzyme_F/UP_NotI_R and DOWN_NotI_F/DOWN_enzyme_R (Supplementary Table S1) for upstream and downstream flanking regions, respectively. The resulting fragments were fused in frame via a NotI digestion site introduced with the indicated primers. The resulting plasmid was transferred via electroporation (400 Ω, 25 μF, 1.2 kV, 0.2 cm Gene Pulser Cuvette: BIORAD) in *B. cereus* ATCC 14579 cells, and plated on BHI agar at 30°C with 10 μg/ml erythromycin (E10) to select for the desired transformants. Two erythromycin resistant colonies were selected and grown overnight in BHI at 30°C in the presence of E10. The resulting culture was diluted (1:200) in fresh LB with E10 and grown o/n at 42°C to select for plasmid integration. Selected strains resulting from a single cross-over integration event were grown overnight in BHI at 30°C to induce double crossover events and subsequently plated and grown at 30°C. Resulting colonies were replica plated on BHI with and without E10 and incubated at 37°C. Colonies sensitive to E10 were selected. PCR analyses (using primers UPFlank_F, DOWNFlank_R, checkINTERNAL_R, check_F, and check_R) (Supplementary Table S1) and DNA sequencing of erythromycin sensitive colonies confirmed the correct internal in-frame deletion of the gene and lack of other mutations in the targeted region.

A double deletion mutant (Δ*cdnL1*/Δ*cdnL2*) was obtained as described above with the exception that the *cdnL2* knock out plasmid was transformed into a *B. cereus* Δ*cdnL1* (BC4714) mutant strain constructed previously (Warda et al., 2016) and 5 μg/ml chloramphenicol was included as selective pressure preventing excision of the chloramphenicol resistance cassette that disrupted the *cdnL1*.

### Heat Treatment

One hundred micro liter of spore suspension containing approximately 10^8^ spores/ml in suspension buffer was transferred, in duplicate, to thin-walled PCR tubes (VWR, The Netherlands). The PCR tubes were kept for 1 min at 4°C followed by a step at 95°C for 45 s and finally cooled for 1 min at 4°C in a thermal cycler (Veriti, Applied Biosystems). The duplicates were pooled and 100 μl of this pooled fraction was used as sample for spore sorting experiments. The same pooled fraction was diluted decimally in suspension buffer and 50 μl samples were used for spore enumeration on BHI plates (in duplicate) and incubated at 30°C up to 3 days with daily enumeration of resulting colonies. For each strain, from the same spore preparation, at least four independent heating experiments were performed.

#### Quantification of Spore Damage

To evaluate the degree of spore damage, the method previously reported by Warda et al. (2016) was used. Briefly, 50 μl of decimally diluted heat treated samples were enumerated in duplicate on BHI plates and BHI plates supplemented with 1.5 and 5.5% salt following incubation at 30°C. To evaluate possible delay in colony formation, colonies were counted after 1, 2, and 7 days (further extension did not affect colony counts). Obtained colony forming units (CFUs) were used to calculate the total damage and fractions of mildly and severely damaged spores as described previously (Warda et al., 2016) according to the following formulas:

$$\begin{array}{c} \%\;Total\;damage=\frac{\left(\mathrm{Number}\;\mathrm{of}\;\mathrm{cf}u\prime s\;\mathrm{BHI})-(\mathrm{Number}\;\mathrm{of}\;\mathrm{cf}u\prime s\;\mathrm{BHI}\;5.5\%\;\mathrm{NaCl}\right)}{\left(\mathrm{Number}\;\mathrm{of}\;\mathrm{cf}u\prime s\;\mathrm{BHI}\right)}\times 100\\ \%\;Mild\;damage=\frac{\left(\mathrm{Number}\;\mathrm{of}\;\mathrm{cf}u\prime s\;\mathrm{BHI}\;1.5\%\;\mathrm{NaCl})-(\mathrm{Number}\;\mathrm{of}\;\mathrm{cf}u\prime s\;\mathrm{BHI}\;5.5\%\;\mathrm{NaCl}\right)}{\left(\mathrm{Number}\;\mathrm{of}\;\mathrm{cf}u\prime s\;\mathrm{BHI}\right)}\times 100\\ \%\;Severe\;damage=\frac{\left(\mathrm{Number}\;\mathrm{of}\;\mathrm{cf}u\prime s\;\mathrm{BHI})\;-(\mathrm{Number}\;\mathrm{of}\;\mathrm{cf}u\prime s\;\mathrm{BHI}\;1.5\%\;\mathrm{NaCl}\right)}{\left(\mathrm{Number}\;\mathrm{of}\;\mathrm{cf}u\prime s\;\mathrm{BHI}\right)}\times 100 \end{array}$$

### Flow Cytometry and Cell/Spore Sorting

Flow cytometry was performed with a FACSAriaIII cell sorter (BD Biosciences) using a fiber launched solid state air-cooled laser operating at 488 nm. Only forward scatter (FCS) and side scatter (SSC) functionality was used. The machine was calibrated using standard Cytometer Setup & Tracking beads and Accudrop beads (BD Biosciences). All parameters were measured using logarithmic amplification. During the procedures a 85 micron nozzle (drop driving frequency was ∼45 kHz/s) was used with flow rate one and during sorting a maximum event rate of 2000 events/s was used. Cells and spores were discriminated from electronic noise using both SSC and FSC. Sorting criteria and gating strategy were based on FSC and SSC populations (data collection equals 50.000 events) excluding remaining doublets. In order to achieve high purity and recovery, the “Single Cell” precision mode (Purity mask 32 and Phase mask 16) was used for sorting. Cells or spores were sorted on solid and in liquid media.

#### Cell Sorting

Five micro liter of an overnight grown culture was diluted in 3 ml of HEPES buffer and loaded into the flow cytometer. Individual vegetative cells of *B. cereus* ATCC 14579 and the mutant derivatives were spotted in duplicate on a single BHI and rice agar plate (according to the scheme in Supplementary Figure S1C) and incubated at 30°C up to 3 days to confirm that growth was not affected in the deletion mutants.

#### Spore Sorting

One hundred micro liter of unheated or heat treated spore suspension (containing non-damaged, damaged and dead spores) was diluted in 1.5 ml of HEPES buffer (pH 7.4) in 5 ml polystyrene falcon tube (BD, USA) and loaded into the flow cytometer. For heat treated spores, a series of 1, 10, and 100 individual spores were sorted either into wells of 384-well plates (Greiner Bio-One, USA) containing 50 μl of BHI or rice broth or on one of the 52 available locations on standard BHI or rice agar plates. The resulting growth data representing three consecutive decimal dilutions were used as input for the MPN quantification method (Oblinger and Koburger, 1975; Jarvis, 2012). For heat treated spores, for each sorting series of 1, 10, or 100 spores approximately 754 replicates were performed for liquid media and at least 520 replicates for solid media (Supplementary Table S2). A single replicate is defined as one well or location on agar plate to which either 1, 10, or 100 spores were sorted. For untreated spores, only single spores were spotted on 188 and 104 locations (Supplementary Table S2) for liquid or solid media, respectively. The resulting plates were incubated at 30°C up to 3 days with daily visual scoring for growth, i.e., colony formation on solid media or appearance of turbidity for liquid media. Wells that were positive for turbidity ranged from OD_600_ 0.2 to 0.3 for rice media (OD_600_ of fresh media 0.16), and in case of BHI values from OD_600_ 0.2 to 0.8 (OD_600_ of fresh media 0.1). The MPN values and their upper and lower limits were calculated using MPN Calculator^1^.

### Model Food Media used in This Study

A rice based medium was prepared according to the method reported previously (Warda et al., 2015) by boiling ready-to-cook pouches filled with 125 g rice produced by the manufacturer (Lassie B.V, The Netherlands) in demineralized water (5:32 w/v) for 45 min. The rice bags were removed and the remaining liquid was allowed to cool down. The method was modified by addition of a centrifugation step [(AVANTI J-25, Beckman Coulter, USA) for 5 min at 16,000 rpm at 22°C] and filtering of the resulting supernatant (Filter paper, Whatman, England) to remove the big particles and improve the clarity of the solution. Finally, the suspension was pooled and autoclaved. Sterile rice broth was stored in the dark until use. For preparation of rice agar plates, 1.5% (w/v) Bacteriological Agar was added prior to a second autoclaving step. The final pH of rice broth was 6.7, while the pH of rice agar plates was 7.

## Results

### Impact of Matrix on the Growth of *B. cereus* Spores

**FIGURE 1:**
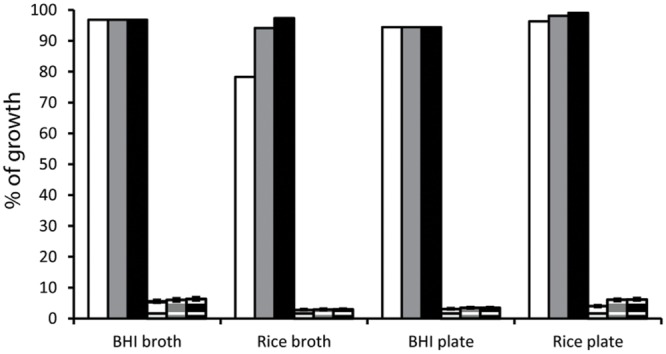
**Percentage of growth from single untreated spores (plain bars) and percentage of growth from heat treated for 45 s at 95°C spores (pattern bars) of *Bacillus cereus* ATCC 14579 after one (white), two (gray) and three (black) days of incubation in liquid (BHI and rice broth) and on solid (BHI and rice agar plates) media.** Values indicated for untreated spores were calculated based on 188 individual spores for recovery in liquid media and 104 individual spores for recovery in solid media. Values for heat treated spores were calculated based on at least 750 wells for each of 1, 10, and 100 spores in liquid media and at least 520 locations for each of 1, 10, and 100 spores on solid media using the MPN based approach. Error bars for heat treated spores represent the lower and upper limits of the MPN values expressed in percentage.

The impact of the liquid and solid media composition on the growth of *B. cereus* spores was evaluated using FCM in combination with single spore sorting. The single untreated *B. cereus* spores were sorted into four different media namely BHI broth, rice broth, BHI agar plates and rice agar plates. Besides BHI, a rice media was selected as this food matrix was shown previously to support growth from *B. cereus* spores on agar plates (Warda et al., 2015) and on Anopore strips (Warda et al., 2015) that resemble growth in broth (den Besten et al., 2007). All four media allowed outgrowth of 94 up to 99% of the sorted spores within 3 days (**Figure 1**; Supplementary Table S2). In rice broth, growth was delayed compared to BHI broth, which was not observed for the corresponding agar media (**Figure 1**). In general, smaller colonies were formed on rice agar plates compared to BHI agar plates for both outgrowing untreated spores (data not shown) and vegetative cells (Supplementary Figure S1). However, after 3 days, the percentage of outgrowing spores on rice agar plates reached 99.1% while on BHI agar plates 94.4% was reached (**Figure 1**).

### Impact of Matrix on the Recovery of Heat Treated *B. cereus* Spores

To allow for high throughput heat treatment of spores, spores were treated in thin-wall tubes in a PCR machine. Using this approach, a 45 s holding time at 95°C resulted in approximately 2 log inactivation and 99% of damaged spores in the surviving population of wild type spores (Supplementary Figure S2). This number is comparable to previous results (91 to above 95%) obtained with capillary tubes in an oil bath (Warda et al., 2015, 2016). In the surviving population, 13% of spores were mildly damaged, whereas 86% were severely damaged. In previous findings, these numbers were 46 and 45%, for mildly and severely damaged spores, respectively (Warda et al., 2016). We previously showed that a *cdnL1* mutant was affected in the ratio between mildly and severely damaged spores (Warda et al., 2016). However, the slightly different heating conditions in the high throughput method resulted in a higher fraction of severely damaged spores in the wild type spores. Using shorter holding times, an increased survival was obtained but again a relatively high percentage of severely damaged spores was observed (data not shown), therefore further experiments were performed using a holding time of 45 s.

**FIGURE 2:**
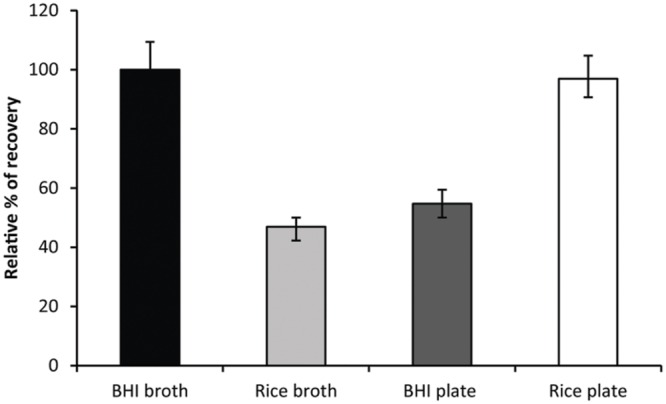
**Recovery of heat treated *B. cereus* ATCC 14579 spores in BHI broth (black), rice broth (light gray), BHI agar plates (dark gray), and rice agar plates (white) after 3 days of incubation.** Values represent the percentage recovery relative to BHI broth (100% corresponds to 6.4% survival of wild type). Error bars represent the lower and upper limits of the MPN values expressed in percentage relative to BHI broth.

To evaluate recovery of sorted heat treated spores in a high throughput format, a combination of single spore sorting with MPN method was applied. Sorting of spores in series of 1, 10, and 100 of heat treated spores at individual locations (well or spot) increased the resolution of the measurements allowing to observe significant differences within expected 2 log inactivation range. Interestingly, for the heat treated spores a comparable recovery in BHI broth and on rice agar plates was observed while rice broth and BHI agar plate supported recovery of approximately 50% of the surviving spores compared to BHI broth (**Figure 2**). This indicated that not only the composition of the media but also its liquid or solid state has an effect on the recovery of the spores.

### Role of Spore Damage Repair Associated Genes in Recovery of Heat Treated *B. cereus* Spores

Previously, genes expressed during germination and outgrowth of heat treated *B. cereus* spores were studied in a transcriptome study resulting in a set of 21 genes that were highly expressed in heat treated spores relative to the reference time point at 10 min but either temporally or not expressed in untreated spores. Further evaluation with qPCR (Warda et al., 2016) to confirm the microarray data resulted in selection of 13 target genes that were downregulated in untreated spores and/or upregulated in heat treated spores with expression ratio below minus two or above two. This selection included the eight genes previously shown to be specifically upregulated during germination and outgrowth of heat damaged *B. cereus* spores, namely BC1312, BC3437, BC3438, BC3921, *cdnL1* (BC4714), BC4834, BC5038, and BC5242 (Warda et al., 2016). A mutant strain in one of those candidate genes (*cdnL1* (BC4714)), a putative transcriptional regulator, was slightly but significantly affected in repair and outgrowth of heat treated *B. cereus* spores (Warda et al., 2016). A paralog of *cdnL1, cdnL2* (BC3648) is encoded on the *B. cereus* ATCC 14579 genome and it was hypothesized that its gene product masked effects on spore damage recovery in the *cdnL1* deletion mutant. Therefore, a *cdnL2* (BC3648) mutant and a combined *cdnL1*/*cdnL2* mutant were included in the present study.

Attempts to construct mutants in BC3438 and BC5038 were unsuccessful. Of the 13 successfully constructed mutants (**Table 1**), four displayed various sporulation defects, mutants either did not sporulate (ΔBC4834), displayed an incomplete sporulation process (ΔBC1312) or the resulting spores were not fully released form the mother cell (ΔBC3437 and ΔBC3921). Therefore, these mutants were excluded from further analysis.

Spores of *B. cereus* ATCC 14579 and its mutant derivatives, were exposed to wet heat treatment for 45 s at 95°C. The reduction in survival of deletion mutants ranged from one up to two log with over 95% of surviving spores being damaged. The fractions of mildly and severely damaged spores were comparable to the wild type (Supplementary Figure S2). The high fraction of damaged spores allows for the assessment of the roles of candidate genes in recovery of heat treated *B. cereus* in different outgrowth conditions, i.e., liquid and solid forms of rice and BHI media.

**FIGURE 3:**
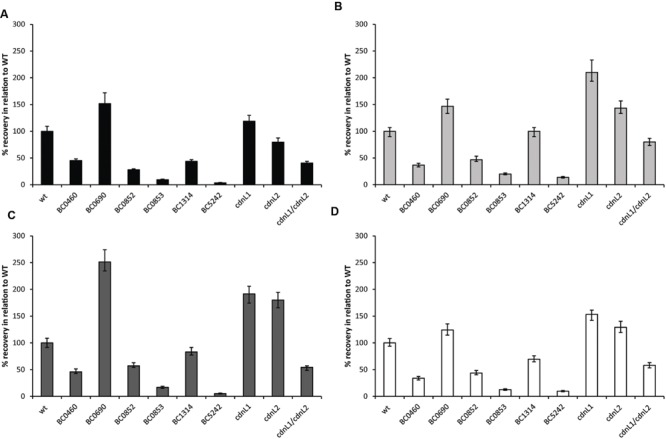
**Recovery of heat treated *B. cereus* ATCC 14579 spores and its mutant derivatives in **(A)** BHI broth, **(B)** rice broth, and on **(C)** BHI agar plates, and **(D)** rice agar plates.** Values are given in percentage relative to the recovery of wild type in given media. Error bars represent the lower and upper limits of the MPN values expressed in percentage relative to the recovery of wild type in given media.

1, 10, and 100 of heat treated spores were sorted either in individual wells of a 384 well plate or onto agar plates resulting in four recovery conditions, namely BHI broth, rice broth, BHI agar plates, or rice agar plates. Deletion mutants ΔBC5242 and ΔBC0853 were highly affected reaching only 3.6 and 9.4% recovery in BHI broth compared to that of wild type spores, respectively (**Figure 3A**). Deletion of ΔBC5242 and ΔBC0853 led to the highest reduction in recovery for all tested media (**Figure 3**) suggesting that effects of these genes on recovery and possibly damage repair were media independent. In contrast, deletion of BC0690 resulted in higher recovery compared to the wild type in both BHI broth (50% increase) and on BHI agar plates (150% increase) (**Figures 3A,C**). Deletion of *cdnL1* (BC4714) resulted in a recovery in BHI broth comparable to that of the wild type, albeit that time to growth was delayed in BHI broth and to a lesser extent on BHI agar plates (Supplementary Figure S3, data not shown). Deletion of *cdnL2* (BC3648) resulted in a slight reduction in recovery compared to wild type in BHI broth, while recovery of the *cdnL1/cdnL2* double mutant (ΔBC4714/ΔBC3648) was reduced by approximately 50% in all tested media compared to wild type (**Figure 3C**). BC0852 and BC0460 mutants displayed a comparable reduction in recovery as the *cdnL1/cdnL2* double mutant. Finally, the recovery of ΔBC1314 depended on the recovery media, in rice broth the recovery was comparable to wild type (**Figure 3B**) while in BHI broth deletion led to over 50% reduction in recovery compared to wild type. Recovery of heat treated spores of all but two (ΔBC0460 and ΔBC0690) deletion mutants was higher on rice agar plates, compared to BHI broth (Supplementary Figure S4). The recovery of those mutant spores improved also in rice broth and on BHI agar plates when compared to relative recovery of the wild type. This suggests that conditions supporting slower growth favor recovery of spores possibly by providing additional time for damage repair.

## Discussion

The capacity of spores to repair damage and grow out is not only affected by the processing conditions, but also by spore history and recovery conditions. Although, several studies report on impact of food components on spore survival and cell growth (Carlin et al., 2000; Choma et al., 2000; Moussa-Boudjemaa et al., 2006), mainly plate counting methods that do not allow for analysis of individual spores have been applied. Moreover, the standard plate counting methods are generally not sensitive enough to show changes within the 10-fold range. In practice, product spoilage may result from a single surviving spore and knowledge on behavior of individual spores can assist in risk evaluation. Here we applied a FCM supported single spore sorting approach in combination with MPN methodology, allowing for evaluation of behavior of individually sorted spores with high resolution for both untreated as well as heat treated spores.

The 45 s heat treatment at 95°C of *B. cereus* wild type and mutant spores resulted in approximately 2 log inactivation, and above 95% damaged spores in the surviving population, which is comparable to previously reported survival and total damage at this temperature (Warda et al., 2015, 2016). Limited information is available on the effect of the recovery media on outgrowth of single damaged spores. In the present study, we focused on the effect of media composition, either liquid or solid state, on the combined process of germination, outgrowth and vegetative growth of individually sorted untreated and heat treated *B. cereus* ATCC 14579 wild type and mutant spores. Firstly, we showed for wild type spores that rice broth was least supporting the growth and recovery of heat treated spores while rice agar plates provided comparable recovery as BHI broth, indicating that not only the composition but also the liquid or solid state of media effects the recovery of heat treated spores. Both heat treated and untreated *B. cereus* spores showed similar recovery when plated on BHI and rice agar plates (Warda et al., 2015). However, the formation of microcolonies from individual spores on Anopore (a porous membrane allowing nutrient transfer that provides surface for spore/cell growth) conditions, which is more close to conditions in a broth (den Besten et al., 2007) were found different for BHI and rice (Warda et al., 2015). More specifically, rice media increased heterogeneity and delayed outgrowth of untreated spores compared to BHI, and also a heat treatment had a limited additional effect on the behavior of surviving spores (Warda et al., 2015). Now we show that outgrowth from untreated single spores was slower in rice based media compared to BHI, but final counts for untreated single sorted spores on rice plates were 99.1% while on BHI plates 94.4%. In line with our previous observations, the time required for colony formation from untreated *B. cereus* spores on rice media was extended compared to BHI, indicating that rice media may contain additional factors delaying germination and/or outgrowth or contain suboptimal concentrations of required components (Warda et al., 2015).

Comparative analysis of wild type and selected mutants lacking genes with putative roles in damage repair, showed different capacity to recover from heat stress compared to wild type. The most pronounced effect was observed for a deletion mutant, lacking a membrane protein with C2C2 zinc finger (BC5242). This mutation resulted in reduction in recovery down to 3.6% of the wild type recovery in BHI broth. The function of BC5242 is unknown, but orthologs of its gene product can be found in many *B. cereus* group strains though not in *B*. *subtilis* 168. In eukaryotes, zinc finger containing proteins function in gene transcription, translation, mRNA trafficking, cytoskeleton organization, epithelial development, cell adhesion, protein folding, chromatin remodeling, and zinc sensing (Laity et al., 2001; Gamsjaeger et al., 2007). In prokaryotes, zinc finger motifs (C4 superfamily) are found in proteins involved in DNA damage recognition, i.e., UvrA, Ada, RecR (Ayora et al., 1997), however, the diversity in functionality of zinc finger carrying proteins and the zinc finger domains does not allow for prediction of a role for BC5242 in *B. cereus*. Notably, BC5242 was not upregulated in vegetative cells of *B. cereus* ATCC 14579 in response to different stresses including cold, ethanol, some disinfectants, and mild acid (Abee et al., 2011).

BC1314 was found to be highly upregulated during germination and outgrowth of heat damaged *B. cereus* spores (Warda et al., 2016). The recovery of ΔBC1314 spores after a heat treatment was decreased with 50% compared to wild type in BHI broth, and on BHI and rice agar plates, albeit less severe for the latter two media, thus suggesting a role of BC1314 in the recovery of heat treated spores. Analysis of the *B. cereus* ATCC 14579 genome sequence suggested that BC1314 (and BC1315) result from a frame-shift mutation in the *phaQ* gene (Supplementary Figure S5). The *B. cereus phaQ* gene is part of a poly-β-hydroxybutyrate (PHB) synthesis cluster, and PHB was previously shown to be accumulated in cells in the form of granules that serve as a carbon and energy source during the late sporulation process in *B. cereus* (Kominek and Halvorson, 1965) and *B*. *megaterium* (Slepecky and Law, 1961). In *B*. *megaterium*, PHB accumulation involves five genes, namely *phaP* (encoding a phasin protein), *phaQ* (encoding a repressor of *phaP* expression), *phaB* (acetoacetyl-CoA reductase), *phaR* and *phaC* (subunits of PHB synthase) (Supplementary Figure S5) (McCool and Cannon, 1999; Lee et al., 2004). Furthermore, in *B*. *thuringiensis* accumulation of PHB via *phaPQRBC* was shown to be under the control of the sporulation transcription factors sigH and Spo0A (Chen et al., 2010). In strains belonging to the *B. cereus* group, orthologs of the *phaPQRBC* system are commonly present, while being absent in *B*. *subtilis* 168, pointing to a special role for this system in the indicated group.

The *cdnL1/cdnL2* double deletion mutant (ΔBC4714/ΔBC3648), lacking genes encoding both CdnL transcriptional regulators present in *B. cereus* ATCC 14579 showed 60% reduction in recovery in BHI broth compared to the wild type. Deletion of *cdnL1* (BC4714) was shown previously to increase the fraction of severely damaged spores in the surviving population after a heat treatment of 1 min at 95°C (Warda et al., 2016). Since the heat treatments applied in the present study led to a dominant fraction of severely damaged spores already in the wild type, we could not observe the increase in percentage of severely damaged spores in Δ*cdnL1* (BC4714). However, outgrowth from heat treated Δ*cdnL1* spores was delayed in BHI broth compared to the wild type spores, eventually reaching comparable recovery efficiency. The Δ*cdnL2* mutant (BC3648) showed lower recovery compared to Δ*cdnL1* (BC4714), and this was most pronounced in liquid media. Nevertheless, both Δ*cdnL1* and Δ*cdnL2* in media other than BHI broth show improved recovery compared to wild type. It remains to be determined whether the observed increase in recovery of the individual *cdnL* mutants could be explained by cross regulation of the counterpart. Both *cdnL1* and *cdnL2* genes are induced in vegetative cells in response to various environmental stresses, including salt and cold stress, whereas acid and oxidative stress specifically induced expression of *cdnL1* and not *cdnL2* (Abee et al., 2011). Our findings suggest partly overlapping functionalities of *cdnL1* and *cdnL2* in recovery and possibly repair of heat damage.

Spores of the ΔBC0690 mutant, lacking a putative PbsX family transcriptional regulator of unknown function, showed higher recovery compared to wild type spores in all tested conditions, with increase of up to 150% on BHI agar plates. Orthologs of BC0690 are commonly found among *B. cereus* group strains, but absent in *B*. *subtilis* 168, pointing possibly to a unique, but up to now unknown role in heat stress survival in *B. cereus* group members.

Deletion of BC0852 and BC0853, both encoding putative quaternary ammonium compound resistance proteins annotated as *sugE*, resulted in reduction in recovery of spores to 9.4 and 28.1% of the wild type in BHI broth, respectively. Orthologs of BC0852 and BC0853 are present in *B. cereus* group strains, while being absent in *B*. *subtilis* 168. Besides BC0852 and BC0853, the *B. cereus* ATCC 14579 genome encodes a second orthologs pair of small multidrug resistance proteins (BC4213 and BC4214) orthologs to *ykkC* (BSU13090) and *ykkD* (BSU13100) of *B*. *subtilis* 168. *ykkC* and *ykkD* are a paired small multidrug resistance (PSMR) members, and their co-expression in *Escherichia coli* led to a multidrug-resistant phenotype (Jack et al., 2000). Still, not all PSMR members have demonstrated drug resistance, e.g., *B*. *subtilis* YvaD/YvaE and YvdR/YvdS, and small multidrug resistance homologs were suggested to be involved in transport of yet unidentified compounds (Bay and Turner, 2009).

In the current study, the applied heat treatment resulted in at least 95% of damaged spores in the surviving wild type and deletion mutant spore populations, based on the fact that these spores were not able to grow out on salt supplemented plates (compared to BHI agar plates). At the moment it cannot be excluded that differences in spore recovery in BHI broth are due to lack of one or more specific proteins in spores of tested deletion mutants that makes them more or less resistant and/or susceptible to heat damage. However, application of rice media and BHI agar plates compared to BHI broth for sorted spores also revealed differences in recovery between media suggesting different requirements for recovery. Particularly deletion of BC0460 or BC0690 resulted in reduced recovery on rice plates while spores of remaining seven deletion mutants showed improved recovery on rice plates compared to BHI broth (Supplementary Figure S4). As the recovery of the various deletion mutants spores appears matrix dependent, this suggests that mutations conceivably affected different type of damage and/or repair targets as was suggested previously by Adams (Adams, 1973). Apparently, high numbers of damaged spores were present in the surviving wild type and mutant spore population, but nevertheless, subtle effects of mutations in putative repair genes were noted, resulting in a shift from the fraction of mildly damaged to the fraction of severely damaged spores (Warda et al., 2016) and in differences in recovery between different media (this study). Still, recovery of heat treated spores is a complex process conceivably involving many different systems, and more studies are required to elucidate the full repertoire of repair systems and the impact of matrix composition and its solid or liquid state on this process.

## Conclusion

We have shown that recovery of heat treated *B. cereus* spores is affected by the matrix composition with highest recovery of wild type spores in BHI broth or on rice agar plates, followed by BHI agar plates and rice broth. The comparative analysis of the wild type and newly constructed deletion mutants provided new insights in the putative role of the deleted genes in the recovery of heat treated *B. cereus* spores.

## Author Contributions

Conceived and designed the experiments: AW, MT, TA, MNG. Performed the experiments: AW, MT. Analyzed the data: AW, MT. Wrote the paper: AW, MT, TA, MNG.

## Conflict of Interest Statement

The authors declare that the research was conducted in the absence of any commercial or financial relationships that could be construed as a potential conflict of interest.

## Footnotes

1 http://www.i2workout.com/mcuriale/mpn/
