# Supplementary material for: Recovery of Heat Treated Bacillus cereus Spores Is Affected by Matrix Composition and Factors with Putative Functions in Damage Repair
Source: Front Microbiol. 2016 Jul 18;7:1096. doi: 10.3389/fmicb.2016.01096 (PMC4947961; doi:10.3389/fmicb.2016.01096)
Supplement: Supplementary file 1 [file Table_1.PDF]

Table S1. Primers used in this study.

Primer	Sequence (5'-3')
KO BC0460_UP_EcoRI_F	TATCGAATTCGTCTTGCCAGCGTCCATCTT
KO BC0460_UP_NotI_R	TCTCGCGGCCGCAATTTTATTTCTCCTCTCAATCTGTT
KO BC0460_DOWN_NotI_F	CGTAGCGGCCGCATGAGGAAATTGCAGAGTAGTATAATAA
KO BC0460_DOWN_SalI_R	ATTTGTCGACGGGCTGGCTGGTGCGATGTTA
KO BC0460_UPFlank_F	GCTCTTCTTCATGCGCTACTTCG
KO BC0460_DOWNFlank_R	GCTTTGGCTGGTGGGATTGTC
BC0460_checkINTERNAL_R	TTCGTCACGCTCAATCCCTTTTT
KO BC0460_check_F	CGGTACGACGGAGCCTGAG
KO BC0460_check_R	TCGATGCCGTCTAAACCAAACTT
KO BC0690_UP_EcoRI_F	ACCAGAATTCCCCGATTGTTATTGCTCCAG
KO BC0690_UP_NotI_R	TTACGCGGCCGCATTCATGTTCCCCCTCCATCCA
KO BC0690_DOWN_NotI_F	ATGAGCGGCCGCGGGAGGAAAAGGAGTGATT
KO BC0690_DOWN_SalI_R	TCAAGTCGACGTTGCCATTATTCTCATCGGTGTTAG
KO BC0690_UPFlank_F	CATTTGCGTATATTCTTGTCATCA
KO BC0690_DOWNFlank_R	TTCGTCGCCTTCCGTTTTTA
BC0690_checkINTERNAL_R	ACTTTTTCTGCTAATTGTTGCTGTGT
KO BC0690_check_F	ATGGAAGTGCATCCGTTACTCTACT
KO BC0690_check_R	CATAAATACCATTAATAAATCCGAAACA
KO BC0852_UP_EcoRI_F	ACTAGAATTCGTTAATAAGCTGTGTTCCATCAA
KO BC0852_UP_NotI_R	GCTCGCGGCCGCATACCTCTCACCTCTTATGACTGAC
KO BC0852_DOWN_NotI_F	CTGTGCGGCCGCCTGCTTAAAAATGGGTTGGTT
KO BC0852_DOWN_SalI_R	AATGGTCGACGCGGGCTAGTAATGAGTGAG
KO BC0852_UPFlank_F	CAGTTTTGCGCCTTGTTCTTTCAG
KO BC0852_DOWNFlank_R	TTTTGTTCTTTTATCGGTTTCTTCACTTT
BC0852_checkINTERNAL_R	ATAACGCCAGCTACAACCATCAC
KO BC0852_check_F	CAAGGGGGAGTGGAGAAAATA
KO BC0852_check_R	GGAAGTATACAAAAATGCGAAGGATG
KO BC0853_UP_EcoRI_F	ATTCGAATTCATTTATGGTTAGATTGTATTTGTTAGA
KO BC0853_UP_NotI_R	ACACGCGGCCGCATTTTTAAGCAGCTCCTTCCACAGT
KO BC0853_DOWN_NotI_F	CACAGCGGCCGCCACTTTCTTAAAGATATATTGCAT
KO BC0853_DOWN_HindIII_R	CACAGTCGACCGTAACACCAGGCATAAT
KO BC0853_UPFlank_F	TAATCCGTTTAATTCTTCTTGTCTTC
KO BC0853_DOWNFlank_R	TCCAGGCCCTATAATGAATCCAGTA
BC0853_checkINTERNAL_R	TCCTATCACACCGACTATTTCACTAA
KO BC0853_check_F	TTTAATACGGCTAATACTTGGTGGCAT
KO BC0853_check_R	TTTCGACGCCCTTTTCCTG
KO BC1312_UP_EcoRI_F	CTCTGAATTCAAGTCTATATTTTATTTTTTCTATTTT
KO BC1312_UP_NotI_R	TCTAGCGGCCGCATTTTGTCCTCCTTCTTTTCCA
KO BC1312_DOWN_NotI_F	TAGTGCGGCCGCCAGCGACAATACTAAAAAAAGAG
KO BC1312_DOWN_SalI_R	AAGCGTCGACAGCACCAATAAACATAAGGAG
KO BC1312_UPFlank_F	GATCAATCACTTTACCTACACTCCCTT
KO BC1312_DOWNFlank_R	TACTTCGAAAGCACTCATCAATAGA
BC1312_checkINTERNAL_R	ACGAGCATACCGTGAGCAATAC
KO BC1312_check_F	TTAACGAGTGGCAGGACAAATG
KO BC1312_check_R	GTGCCGACAACTGATAATACAAGAA
KO BC1314_UP_EcoRI_F	TACAGAATTCCGCGCTCCCAATCTTCACGA
KO BC1314_UP_NotI_R	CACAGCGGCCGCATGATTGTGCATCACCTCA
KO BC1314_DOWN_NotI_F	CACAGCGGCCGCCAGAGTAATCTGCGTTCACAATA
KO BC1314_DOWN_SalI_R	CCTAGTCGACACACATCCTTATGAGTAGTCCAGTTC
KO BC1314_UPFlank_F	CTAATAATTCTTCCTTCTTCTGCTTC
KO BC1314_DOWNFlank_R	TTGTTCTGCACCTGGAACTAATAAT
BC1314_checkINTERNAL_R	TTTTTGGCATGGAGTTTGGTTG
KO BC1314_check_F	TCAAATCTAGAACGCTGCCCATC
KO BC1314_check_R	CCTTCCATTTGTCCTGCCACT
KO BC3437_UP_BamHI_F	TCTCGGATCCAAGCGGTTGCCATTTTACTC
KO BC3437_UP_NotI_R	CATGGCGGCCGCTAACATCTTATTTTCCTCCTTTTTT
KO BC3437_DOWN_NotI_F	GTCAGCGGCCGCATAAATGGAATAAGTAGTA
KO BC3437_DOWN_SalI_R	TGCCGTCGACGCCGCCAAACACGTAGACAAATAATAG
KO BC3437_UPFlank_F	GCTGATGCTTTCCAATGTGA
KO BC3437_DOWNFlank_R	TTACATCCGTAAAGAGTCGCAC
BC3437_checkINTERNAL_R	TCCATCCCGTTACCCGCTGAA
KO BC3437_check_F	CTATGAGATTACCCGCCACCTGA
KO BC3437_check_R	TCGTCTTTCCCGCTCCATTTGA

Table S1. (Continued) Primers used in this study.

Primer	Sequence (5'-3')
KO BC3648_UP_BamHI_F	ATCAGAATTCGGGATGCTTACAATTATGCTTACG
KO BC3648_UP_NotI_R	TTACTGCGGCCGCGAAACAAATAATCCACCTCCATA
KO BC3648_DOWN_NotI_F	ACATCGCGGCCGCAGCTTTTGTTAAGGTTCATTACA
KO BC3648_DOWN_HindIII_R	TTGTTAAGCTTCACCGCCATATTTATCTGTTCG
KO BC3648_UPFlank_F	AAATTGAAAATGACACCAGAAGAAGTT
KO BC3648_DOWNFlank_R	TGAAAGCAGAGAAACGAAGAAGTGT
BC3648_checkINTERNAL_R	CTGCTCCCTGCATTGGATAAACA
KO BC3648_check_F	TGCCTTAACAAAAACACATCACTACT
KO BC3648_check_R	TCTCTTTCATCCTTACGGCTCCTC
KO BC3921_UP_EcoRI_F	GTATGAATTCATCGCATTAATTTCCGTCTGTCTAT
KO BC3921_UP_NotI_R	TTTGGCGGCCGCATTACATCACATCCTTTTGT
KO BC3921_DOWN_NotI_F	ACCTGCGGCCGCCTTAAAAAATAAAAAAACAGC
KO BC3921_DOWN_SalI_R	GATTGTCGACGGATGAGATGGTAGATGTAA
KO BC3921_UPFlank_F	TAATTCTTCCTTTCGTATCCAGTTCT
KO BC3921_DOWNFlank_R	GCTAGCTATAATTTTGTTTGTTGCCTTGA
BC3921_checkINTERNAL_R	TGCCAAATATAAAAATCACCATCAG
KO BC3921_check_F	CGCCCTATAATTTCGTTGTCCT
KO BC3921_check_R	ATGCTCGCTCGAATGAGAAGTTAGAGAA
KO BC4834_UP_EcoRI_F	ATTAGAATTCTTGTTGTTTTACTTCATACGA
KO BC4834_UP_NotI_R	CCATGCGGCCGCTGATTCAATGTCTTTCTTCCTTTC
KO BC4834_DOWN_NotI_F	GGGTGCGGCCGCTTACGAATAAGGGGGAAGAG
KO BC4834_DOWN_SalI_R	GAGAGTCGACAGAATGGAGGAAAAAGGAGAAC
KO BC4834_UPFlank_F	CGTCTTCCATTTCTAGCACCATA
KO BC4834_DOWNFlank_R	TTAAACCCAGAGTCTATTCAAACATT
BC4834_checkINTERNAL_R	TAATAAGTCTAAAATTTTTCTCCTTGTTG
KO BC4834_check_F	GCTATAGTGTATCATGTGTTTCAAGTCT
KO BC4834_check_R	AGTAGCAGTAGTATCCCGTTATTCATCA
KO BC5242_UP_EcoRI_F	ATATGAATTCAGTAAAGTGCTGCATGTAACCTG
KO BC5242_UP_NotI_R	TGACGCGGCCGCATTTAATTCACTCCCCAC
KO BC5242_DOWN_NotI_F	TTTAGCGGCCGCATATATATATTACATTAG
KO BC5242_DOWN_SalI_R	AATCGTCGACTCAATTTTCACTTTCTCGCTCTGTTC
KO BC5242_UPFlank_F	CGAACGATGGATTCCACTTTATCTAC
KO BC5242_DOWNFlank_R	TTCATCAGAAGAAATGCTTACATACT
BC5242_checkINTERNAL_R	CATTTTCACTGCCACAACCTCT
KO BC5242_check_F	GCTTAAAAGAAATGATTGCTCCAG
KO BC5242_check_R	ACGATTACTTCTACTTTACTCCCACACT
