# Supplementary material for: Recovery of Heat Treated Bacillus cereus Spores Is Affected by Matrix Composition and Factors with Putative Functions in Damage Repair
Source: Front Microbiol. 2016 Jul 18;7:1096. doi: 10.3389/fmicb.2016.01096 (PMC4947961; doi:10.3389/fmicb.2016.01096)
Supplement: Supplementary file 2 [file Table_2.PDF]

Table S2: Total number of locations with sorted one, 10 or 100 spores and resulting number of locations showing growth in tested media with (A) or without (B) heat treatment. For heat treated spores MPN values are given along with upper and lower limits and conversion to %. MPN numbers are expressed per well or spot on agar plate.

(A)

Recovery media	Strain	1 spore per location		10 spore per location		100 spore per location		MPN values			MPN in %			MPN in % relative to wild type		
		Total number of locations sorted	Total number of locations with grow	Total number of locations sorted	Total number of locations with grow	Total number of locations sorted	Total number of locations with grow	MPN	upper limit	lower limit	MPN	upper limit	lower limit	value	st dev +	st dev -
BHI broth	wild type	753	78	751	333	740	738	**0.064**	0.07	0.058	6.4	0.6	0.6	100.0	9.4	9.4
	ΔcdnL1/cdnL2	754	23	750	186	744	678	**0.026**	0.028	0.024	2.6	0.2	0.2	40.6	3.1	3.1
	ΔcdnL2	753	39	753	296	754	751	**0.051**	0.056	0.047	5.1	0.5	0.4	79.7	7.8	6.3
	ΔcdnL1	754	62	752	392	754	754	**0.076**	0.083	0.069	7.6	0.7	0.7	118.8	10.9	10.9
	ΔBC5242	754	3	753	17	748	155	**0.0023**	0.0027	0.002	0.23	0.04	0.03	3.6	0.6	0.5
	ΔBC1314	754	33	754	180	754	704	**0.028**	0.03	0.026	2.8	0.2	0.2	43.8	3.1	3.1
	ΔBC0853	754	4	754	48	739	332	**0.006**	0.0067	0.0054	0.6	0.07	0.06	9.4	1.1	0.9
	ΔBC0852	754	15	754	139	739	608	**0.018**	0.019	0.017	1.8	0.1	0.1	28.1	1.6	1.6
	ΔBC0690	754	88	753	456	754	754	**0.097**	0.11	0.089	9.7	1.3	0.8	151.6	20.3	12.5
	ΔBC460	754	16	754	112	754	543	**0.029**	0.031	0.027	2.9	0.2	0.2	45.3	3.1	3.1
Rice broth	wild type	754	28	754	216	754	704	**0.03**	0.032	0.027	3	0.2	0.3	100.0	6.7	10.0
	ΔcdnL1/cdnL2	754	19	754	176	754	681	**0.024**	0.026	0.022	2.4	0.2	0.2	80.0	6.7	6.7
	ΔcdnL2	754	40	754	269	754	741	**0.043**	0.047	0.04	4.3	0.4	0.3	143.3	13.3	10.0
	ΔcdnL1	754	44	754	364	754	751	**0.063**	0.07	0.058	6.3	0.7	0.5	210.0	23.3	16.7
	ΔBC5242	754	9	754	40	754	241	**0.0041**	0.0046	0.0037	0.41	0.05	0.04	13.7	1.7	1.3
	ΔBC1314	754	15	754	196	754	716	**0.03**	0.032	0.027	3	0.2	0.3	100.0	6.7	10.0
	ΔBC0853	1131	7	1131	64	1131	515	**0.006**	0.0066	0.0056	0.6	0.06	0.04	20.0	2.0	1.3
	ΔBC0852	754	14	754	93	754	577	**0.014**	0.016	0.013	1.4	0.2	0.1	46.7	6.7	3.3
	ΔBC0690	754	49	754	243	754	747	**0.044**	0.048	0.04	4.4	0.4	0.4	146.7	13.3	13.3
	ΔBC460	754	16	754	84	754	502	**0.011**	0.012	0.01	1.1	0.1	0.1	36.7	3.3	3.3
BHI plate	wild type	3615	128	1040	314	520	500	**0.035**	0.038	0.032	3.5	0.3	0.3	100.0	8.6	8.6
	ΔcdnL1/cdnL2	520	16	1040	181	520	434	**0.019**	0.02	0.017	1.9	0.1	0.2	54.3	2.9	5.7
	ΔcdnL2	1668	109	1040	503	520	514	**0.063**	0.068	0.058	6.3	0.5	0.5	180.0	14.3	14.3
	ΔcdnL1	520	35	1040	530	520	514	**0.067**	0.072	0.061	6.7	0.5	0.6	191.4	14.3	17.1
	ΔBC5242	520	3	1040	11	520	90	**0.0018**	0.0022	0.0015	0.18	0.04	0.03	5.1	1.1	0.9
	ΔBC1314	520	18	1040	258	520	493	**0.029**	0.032	0.027	2.9	0.3	0.2	82.9	8.6	5.7
	ΔBC0853	520	1	1040	75	520	220	**0.0059**	0.0066	0.0052	0.59	0.07	0.07	16.9	2.0	2.0
	ΔBC0852	520	9	1040	196	520	452	**0.02**	0.022	0.019	2	0.2	0.1	57.1	5.7	2.9
	ΔBC0690	520	48	1040	607	520	520	**0.088**	0.096	0.082	8.8	0.8	0.6	251.4	22.9	17.1
	ΔBC460	520	10	1040	159	520	418	**0.016**	0.018	0.015	1.6	0.2	0.1	45.7	5.7	2.9
Rice plate	wild type	3615	220	1040	482	505	503	**0.062**	0.067	0.058	6.2	0.5	0.4	100.0	8.1	6.5
	ΔcdnL1/cdnL2	520	21	1040	317	520	504	**0.036**	0.039	0.033	3.6	0.3	0.3	58.1	4.8	4.8
	ΔcdnL2	1668	137	1040	578	520	518	**0.08**	0.087	0.074	8	0.7	0.6	129.0	11.3	9.7
	ΔcdnL1	520	40	1040	643	520	520	**0.095**	0.1	0.088	9.5	0.5	0.7	153.2	8.1	11.3
	ΔBC5242	520	4	1040	65	520	232	**0.006**	0.0068	0.0054	0.6	0.08	0.06	9.7	1.3	1.0
	ΔBC1314	520	23	1040	366	520	513	**0.043**	0.047	0.04	4.3	0.4	0.3	69.4	6.5	4.8
	ΔBC0853	520	6	1040	82	520	278	**0.0078**	0.0087	0.007	0.78	0.09	0.08	12.6	1.5	1.3
	ΔBC0852	520	20	1040	263	520	478	**0.027**	0.03	0.025	2.7	0.3	0.2	43.5	4.8	3.2
	ΔBC0690	520	39	1040	559	520	520	**0.077**	0.084	0.071	7.7	0.7	0.6	124.2	11.3	9.7
	ΔBC460	520	4	1040	190	520	464	**0.021**	0.023	0.019	2.1	0.2	0.2	33.9	3.2	3.2

(B)

Recovery media	Strain	1 spore per location		% growth	% growth relative to wild type
		Total number of locations sorted	Total number of locations with grow		
BHI broth	wild type	188	182	96.8	100.0
	ΔcdnL1/cdnL2	188	184	97.9	101.1
	ΔcdnL2	188	182	96.8	100.0
	ΔcdnL1	188	178	94.7	97.8
	ΔBC5242	188	171	91.0	94.0
	ΔBC1314	188	177	94.1	97.3
	ΔBC0853	188	178	94.7	97.8
	ΔBC0852	188	177	94.1	97.3
	ΔBC0690	188	183	97.3	100.5
	ΔBC460	188	177	94.1	97.3
Rice broth	wild type	189	184	97.4	100.0
	ΔcdnL1/cdnL2	188	179	95.2	97.8
	ΔcdnL2	189	179	94.7	97.3
	ΔcdnL1	189	181	95.8	98.4
	ΔBC5242	189	170	89.9	92.4
	ΔBC1314	189	181	95.8	98.4
	ΔBC0853	189	177	93.7	96.2
	ΔBC0852	189	184	97.4	100.0
	ΔBC0690	189	179	94.7	97.3
	ΔBC460	189	185	97.9	100.5
BHI plate	wild type	108	102	94.4	100.0
	ΔcdnL1/cdnL2	208	201	96.6	102.3
	ΔcdnL2	108	104	96.3	102.0
	ΔcdnL1	104	98	94.2	99.8
	ΔBC5242	104	101	97.1	102.8
	ΔBC1314	104	103	99.0	104.9
	ΔBC0853	104	100	96.2	101.8
	ΔBC0852	104	97	93.3	98.8
	ΔBC0690	104	103	99.0	104.9
	ΔBC460	104	102	98.1	103.8
Rice plate	wild type	108	107	99.1	100.0
	ΔcdnL1/cdnL2	208	202	97.1	98.0
	ΔcdnL2	108	107	99.1	100.0
	ΔcdnL1	104	101	97.1	98.0
	ΔBC5242	104	103	99.0	100.0
	ΔBC1314	104	102	98.1	99.0
	ΔBC0853	104	102	98.1	99.0
	ΔBC0852	104	100	96.2	97.1
	ΔBC0690	104	101	97.1	98.0
	ΔBC460	104	104	100.0	100.9
