# Supplementary material for: Recovery of Heat Treated Bacillus cereus Spores Is Affected by Matrix Composition and Factors with Putative Functions in Damage Repair
Source: Front Microbiol. 2016 Jul 18;7:1096. doi: 10.3389/fmicb.2016.01096 (PMC4947961; doi:10.3389/fmicb.2016.01096)

Figure S1. Colonies originating from single vegetative cells of *B. cereus* ATCC 14579 and its mutant derivatives spotted in duplicate on (A) BHI and (B) rice agar plates incubated overnight at 30°C. (C) Schema of spotting.

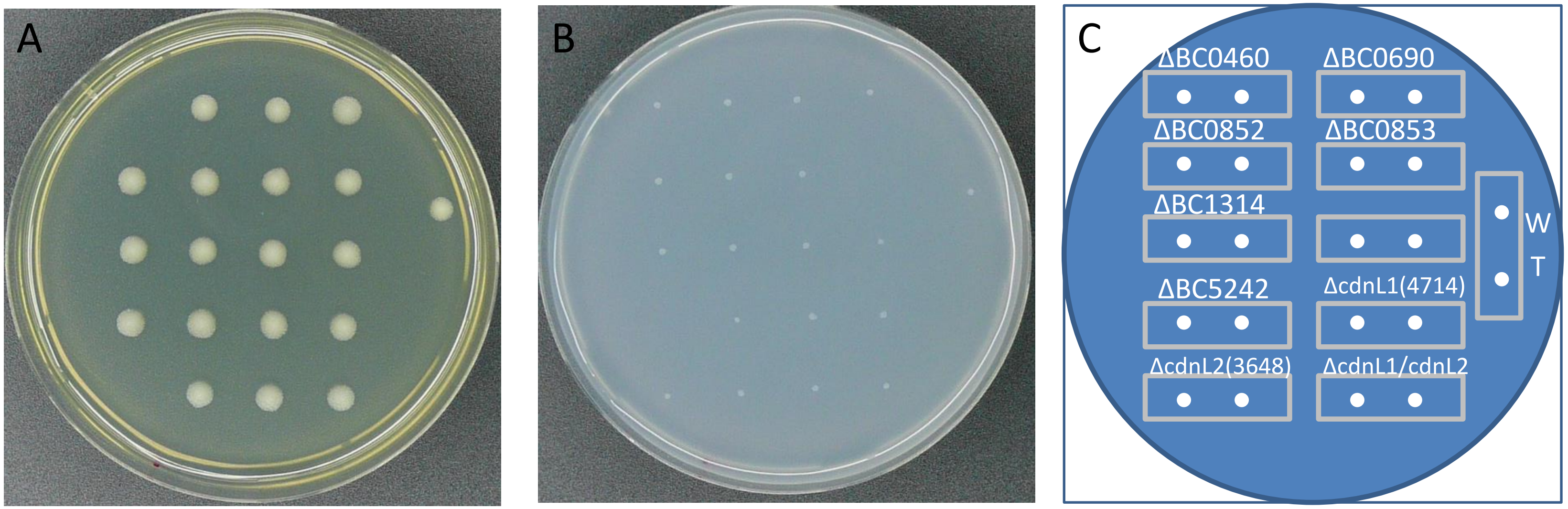

Supplement: Supplementary file 3 [file Image_1.PDF]
