# Supplementary material for: Recovery of Heat Treated Bacillus cereus Spores Is Affected by Matrix Composition and Factors with Putative Functions in Damage Repair
Source: Front Microbiol. 2016 Jul 18;7:1096. doi: 10.3389/fmicb.2016.01096 (PMC4947961; doi:10.3389/fmicb.2016.01096)

Figure S2. Effect of a 45 s heat treatment at 95°C on spores of *B. cereus* ATCC 14579 and its mutant derivatives. (A) Survival and (B) fractions of mildly (light grey) and severely (dark grey) damaged spores among the survivals. Combined mild and severe damage represent total damaged among the survivals. * indicate values significantly (P-value <0.05) different from wild type.

(A)

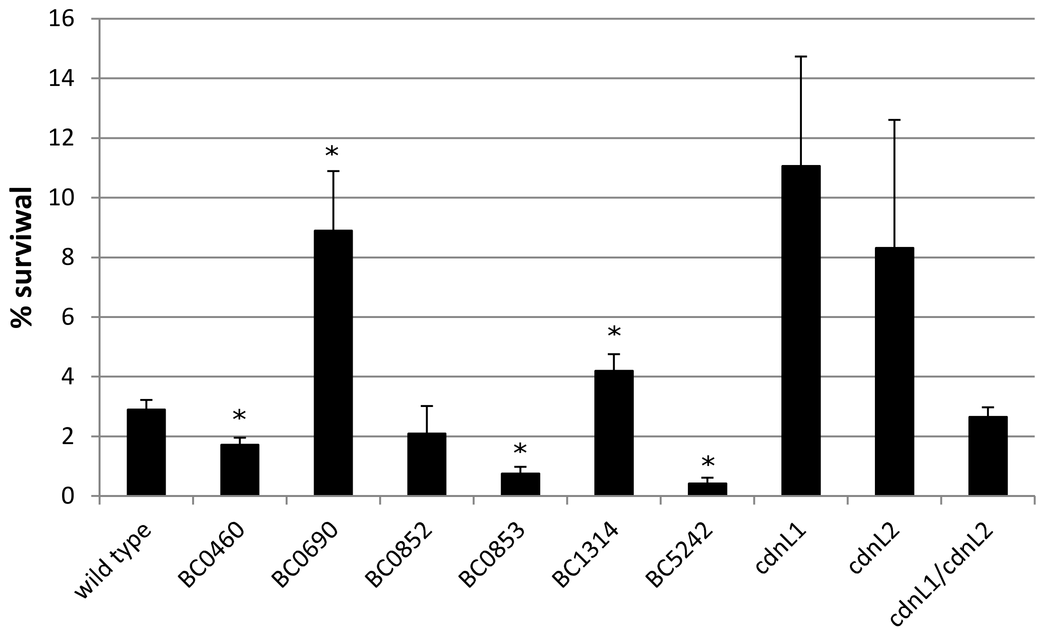

(B)

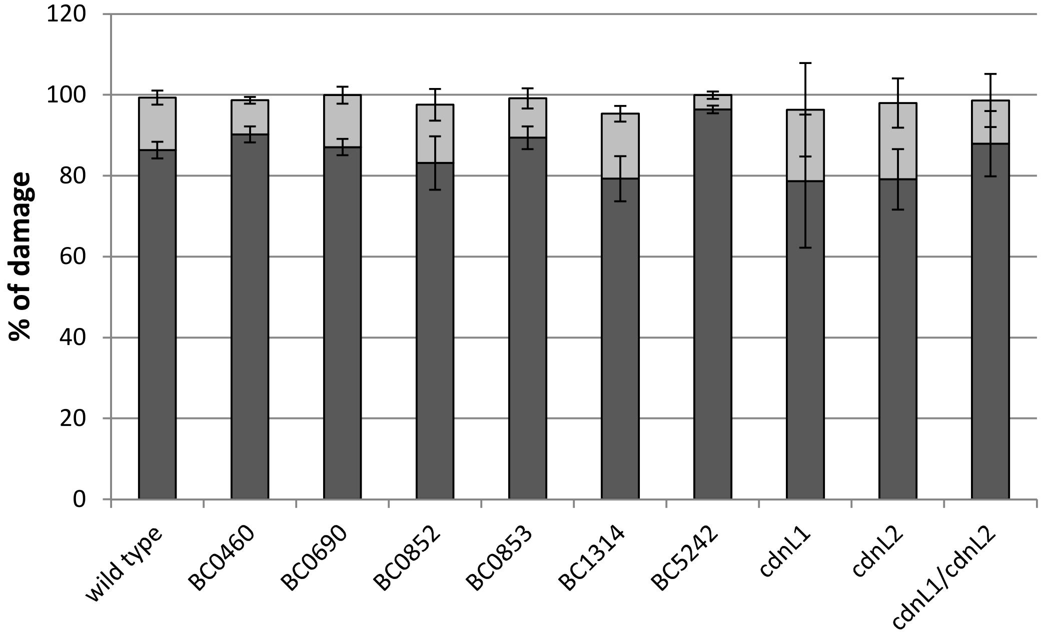

Supplement: Supplementary file 4 [file Image_2.PDF]
