# Supplementary material for: Recovery of Heat Treated Bacillus cereus Spores Is Affected by Matrix Composition and Factors with Putative Functions in Damage Repair
Source: Front Microbiol. 2016 Jul 18;7:1096. doi: 10.3389/fmicb.2016.01096 (PMC4947961; doi:10.3389/fmicb.2016.01096)

Figure S3. Recovery of heat treated *B. cereus* ATCC 14579 spores and its mutant derivatives in BHI broth after one, two and three days incubation. Values are given in percentage relative to the recovery of wild type in BHI broth after three day incubation.

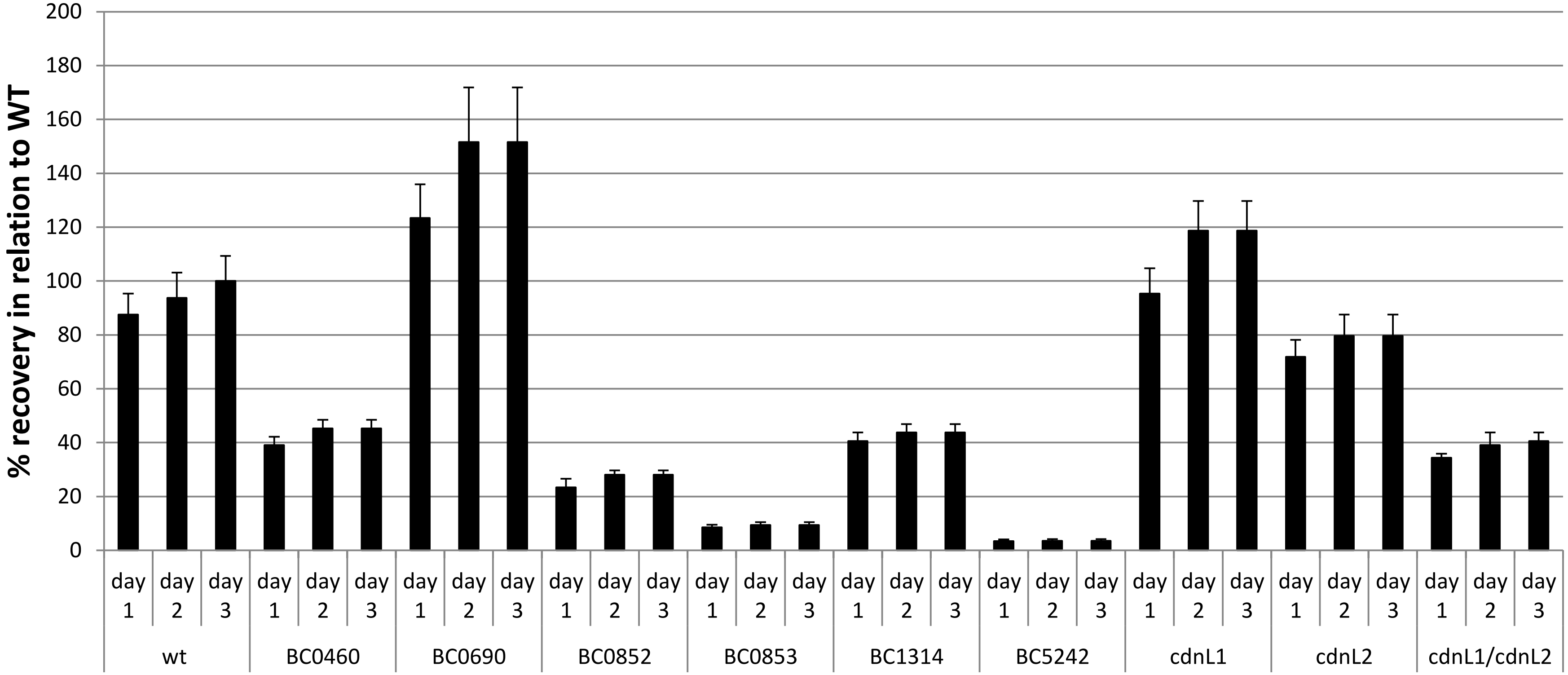

Supplement: Supplementary file 5 [file Image_3.PDF]
