# Supplementary material for: Recovery of Heat Treated Bacillus cereus Spores Is Affected by Matrix Composition and Factors with Putative Functions in Damage Repair
Source: Front Microbiol. 2016 Jul 18;7:1096. doi: 10.3389/fmicb.2016.01096 (PMC4947961; doi:10.3389/fmicb.2016.01096)

Figure S4. Recovery of heat treated *B. cereus* ATCC 14579 spores and its mutant derivatives in BHI broth (black), rice broth (dark grey) and on BHI agar plates (light grey), rice agar plates (white). Values are given in percentage relative to the recovery of a given strains in BHI broth after three day incubation.

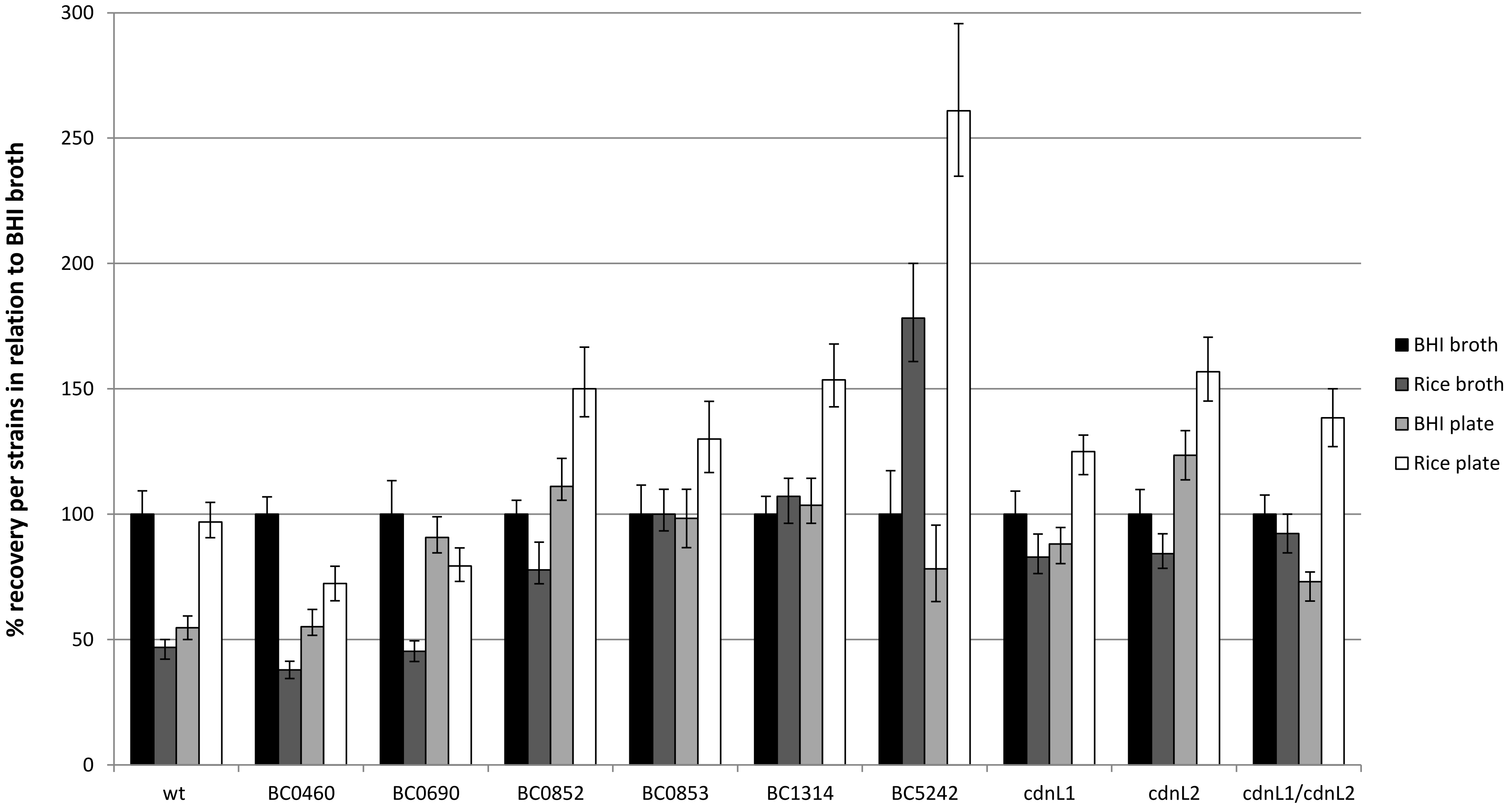

Supplement: Supplementary file 6 [file Image_4.PDF]
