# Supplementary material for: Recovery of Heat Treated Bacillus cereus Spores Is Affected by Matrix Composition and Factors with Putative Functions in Damage Repair
Source: Front Microbiol. 2016 Jul 18;7:1096. doi: 10.3389/fmicb.2016.01096 (PMC4947961; doi:10.3389/fmicb.2016.01096)

**Figure S5. Gene organization and gene context of *phaPQRBC* in *B. cereus* ATCC 14579, *B. cereus* ATCC 10987 and *B. megaterium* QM B1551.** Colour coding for encoded proteins: pink, SASP; white, pseudogene; grey, hypothetical protein.

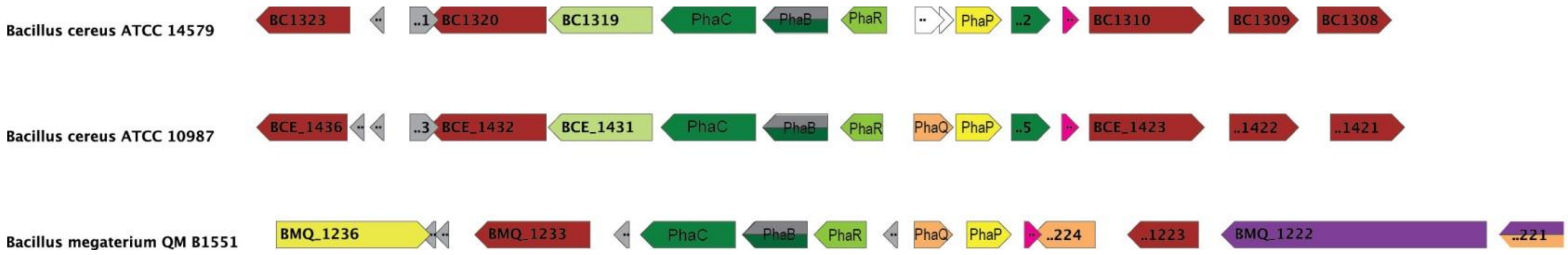

Supplement: Supplementary file 7 [file Image_5.PDF]
